# OsbZIP18 Is a Positive Regulator of Phenylpropanoid and Flavonoid Biosynthesis under UV-B Radiation in Rice

**DOI:** 10.3390/plants13040498

**Published:** 2024-02-10

**Authors:** Xueqing Liu, Ziyang Xie, Jiajun Xin, Shiqing Yuan, Shuo Liu, Yangyang Sun, Yuanyuan Zhang, Cheng Jin

**Affiliations:** 1 School of Breeding and Multiplication (Sanya Institute of Breeding and Multiplication), Hainan University, Sanya 572025, China; 2 School of Tropical Agriculture and Forestry, Hainan University, Haikou 570228, China; 3 Sanya Research Institute of Hainan Academy of Agricultural Sciences, Sanya 572025, China

**Keywords:** UV-B radiation, metabolites, phenylpropanoid and flavonoids biosynthesis, regulator, rice

## Abstract

In plants exposed to ultraviolet B radiation (UV-B; 280–315 nm), metabolic responses are activated, which reduce the damage caused by UV-B. Although several metabolites responding to UV-B stress have been identified in plants, the accumulation of these metabolites at different time points under UV-B stress remains largely unclear, and the transcription factors regulating these metabolites have not been well characterized. Here, we explored the changes in metabolites in rice after UV-B treatment for 0 h, 6 h, 12 h, and 24 h and identified six patterns of metabolic change. We show that the rice transcription factor OsbZIP18 plays an important role in regulating phenylpropanoid and flavonoid biosynthesis under UV-B stress in rice. Metabolic profiling revealed that the contents of phenylpropanoid and flavonoid were significantly reduced in *osbzip18* mutants compared with the wild-type plants (WT) under UV-B stress. Further analysis showed that the expression of many genes involved in the phenylpropanoid and flavonoid biosynthesis pathways was lower in *osbzip18* mutants than in WT plants, including *OsPAL5*, *OsC4H*, *Os4CL*, *OsCHS*, *OsCHIL2*, and *OsF3H.* Electrophoretic mobility shift assays (EMSA) revealed that OsbZIP18 bind to the promoters of these genes, suggesting that OsbZIP18 function is an important positive regulator of phenylpropanoid and flavonoid biosynthesis under UV-B stress. In conclusion, our findings revealed that OsbZIP18 is an essential regulator for phenylpropanoid and flavonoid biosynthesis and plays a crucial role in regulating UV-B stress responses in rice.

## 1. Introduction

With the depletion of the stratospheric ozone layer, an increasing amount of ultraviolet-B (UV-B) irradiation (280–315 nm) reaches the Earth’s surface and the surfaces of plants. Natural levels of UV-B irradiation act as an environmental regulator that controls gene expression and plant growth and development [1,2]. However, excessive UV-B irradiation can cause damage to macromolecules, such as proteins, nucleic acids, and lipids, due to the absorption of the energy-rich irradiation [1,3,4]. Numerous studies have shown that UV-B irradiation reduces the yield indicators of rice, including the number of ears, number of grains, and grain weight, by inhibiting the growth, development, and physiological activities of rice [5,6,7]. Therefore, it is crucial to understand how rice protects itself against the potentially harmful effects of UV-B irradiation.

UV-B irradiation is a significant factor that enhances the production of defense-related secondary metabolites in plants [8,9]. In recent years, it has been demonstrated that certain plant metabolites, including flavonoids [10,11,12,13], phenolics [14,15], ascorbate [16,17], and tocopherol [18], actively protect plants against harmful UV-B radiation. Flavonoids, especially glycosylated flavonoids and phenylacylated flavonols, have attracted considerable attention as antioxidants to protect against UV-B damage from early plants to green plants throughout evolution [19]. Several studies have reported that UV-B irradiation induces the accumulation of flavonoids and the upregulation of genes in the phenylpropanoid pathway, which serve as protective mechanisms against UV-B damage [20,21,22,23]. There is evidence indicating that UV-B induces the biosynthesis of flavonoids with higher hydroxylation levels in *Populus trichocarpa* and *Petunia hybrida* [24,25]. However, the effects of UV-B irradiation at different times on rice metabolism are unknown.

Amino acids are major primary metabolites in plants. Apart from their protein synthesis, they can also undergo catabolism to form intermediates of the tricarboxylic acid (TCA) cycle, which are essential for generating energy [26,27]. Previous studies have found that several amino acids, including proline, serine, leucine, isoleucine, glutamate, and lysine, accumulate in plants after being exposed to intense UV-B irradiation [28,29,30]. These amino acids are closely associated to other metabolic pathways that regulate amino acid metabolism and defense responses. As such, BCAAs can provide electrons directly to the electron transport chain via the electron transfer flavoprotein complex. They can also indirectly contribute because their catabolic products directly enter the TCA cycle [31]. The catabolism of lysine into acetyl-CoA can likely help supplement the TCA cycle with energy to compensate for the energy lost in *G. uralensis* leaves due to UV-B stress [32]. The aromatic amino acids can be converted to numerous secondary metabolites, such as isoquinoline, indole alkaloids, phenylpropanoids, glucosinolates, and auxin, which can protect plants from various stresses [14,33,34,35,36,37,38]. These results suggest that UV-B irradiation has significantly disrupted primary metabolism in rice. It would be beneficial to investigate possible changes in rice metabolism in response to UV-B irradiation, primarily focusing on primary metabolites and their potential connection to secondary metabolism.

Molecularly, UV-B light with photomorphogenesis is effective in triggering differential gene expression in *Arabidopsis thaliana*. In response to UV-B, the UV RESISTANCE LOCUS8 (UVR8) rapidly interacts with the multifunctional E3 ubiquitin ligase known as CONSTITUTIVE PHOTOMORPHOGENESIS1 (COP1) [39,40]. This interaction plays a role in promoting UV-B-induced photomorphogenesis by regulating protein stability [39,40,41]. COP1 degrades two multi-functional UV-B signaling repressors, known as REPRESSOR OF UV-B PHOTOMORPHOGENESIS (RUP1) and RUP2. Ultimately, COP1 stabilizes the central transcription factor ELONGATED HYPOCOTYL5 (HY5), which promotes photomorphogenesis [41,42,43,44]. The expression of numerous genes involved in metabolic pathways, including phenylpropanoid and flavonoid biosynthesis [44,45], anthocyanin [46], chlorophyll, and carotenoid biosynthesis [47], has been proven to be regulated by it. UV-B also induces the expression of other transcription factors in plants. For example, the transcription factor MYB13 in *Arabidopsis* promotes the accumulation of flavonoids by regulating the expression of flavonoid synthesis pathway genes [48]. The B-BOX transcription factor, BBX11, whose expression is induced by UV-B, protects plants from UV-B damage by regulating the accumulation of photoprotective phenols and antioxidants [49]. In rice, OsBBX14 directly interacts with OsHY5 through its second B-box domain to activate anthocyanin biosynthesis genes, *OsC1* or *OsB2* [50,51]. *OsbZIP18*, a HY5 homologous gene in rice, has been identified as a positive regulator of branched chain amino acids (BCAAs) biosynthesis, including valine, leucine, and isoleucine, as well as serotonin biosynthesis, under UV-B irradiation [52,53]. However, whether OsbZIP18 is involved in regulating phenylpropanoid and flavonoid metabolites accumulation under UV-B stress remains unclear.

In this study, metabolomics was employed to uncover potential response mechanisms to UV-B irradiation. Metabolites extracted from leaves were analyzed using liquid chromatography–mass spectrometry (LC/MS). The statistical assessment of changes in metabolites was conducted through principal component analysis (PCA) and dynamic change analysis to gain insights into the cellular metabolism associated with the duration of UV-B irradiation in the plant. We compared the metabolome of both the ZH11 and *osbzip18* mutants under various durations of UV-B treatment. The results of the Kyoto Encyclopedia of Genes and Genomes (KEGG) enrichment analysis revealed significant enrichment in the biosynthesis of phenylpropanoid and flavonoid. Gene expression analysis revealed a decrease in the expression levels of *OsPAL5*, *OsC4H*, *Os4CL*, *OsCHS*, *OsCHIL2*, and *OsF3H*, in *osbzip18* mutants under UV-B irradiation. EMSA assays confirmed OsbZIP18 could directly bind to the ACGT element in the promoters of these genes.

## 2. Results

### 2.1. Metabolic Profile of Rice Leaves under Different Periods of UV-B Irradiation Treatment

**Figure 1:**
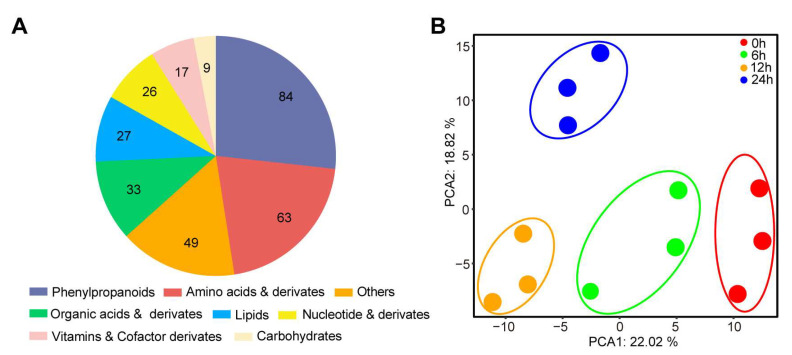
Comparison of rice metabolism under different UV-B duration treatments. (**A**) The composition and classification of metabolites are known. (**B**) Principal component analysis (PCA) of known metabolites under different UV-B duration treatments. Three biological replicates were taken for the analyses from every treatment (n = 3), and each replicate was mixed with eight different plants.

To investigate the effects of UV-B irradiation on the metabolome of rice, the ZH11 variety was treated with UV-B irradiation for different durations. We utilized a broadly targeted metabolomics approach to construct a metabolome database of rice leaves under UV-B irradiation treatment, and then we detected the metabolites of the samples using the QTrap system (QTRAP 6500, AB SCIEX, Toronto, ON, Canada). A total of 308 metabolites were identified by standards and databases, which can be classified into 8 different groups, including 84 phenylpropanoids, 63 amino acids and their derivatives, 33 organic acids and their derivatives, 27 lipids, 26 nucleotides and their derivatives, 17 vitamins and cofactor derivatives, 9 carbohydrates, and 49 other compounds (Figure 1A). Principal component analysis (PCA) was performed to evaluate the overall differences between samples under the unsupervised model. PCA could clearly differentiate between the samples with different duration of UV-B irradiation from the control samples (Figure 1B). The control group’s data points showed a high concentration, suggesting that the collection process was highly repeatable. Furthermore, based on the first principal component (PC1) and the second principal component (PC2), the four groups of UV-B irradiation samples were divided into different regions, and the interpretation rate of the first principal component PCA1 was 22.02% and that of the second principal component PCA2 was 18.82% (Figure 1B). These data suggest that the different UV-B irradiation durations significantly affect metabolites accumulation in rice leaves.

### 2.2. Temporal Analysis of Metabolites in Rice Leaves under Different UV-B Duration Treatment

**Figure 2:**
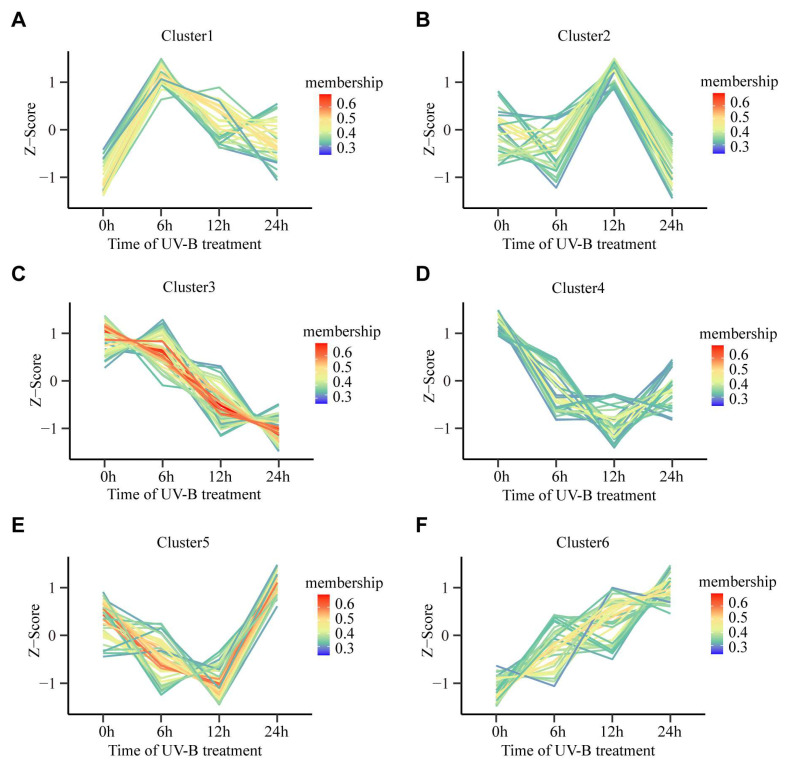
Different patterns of metabolite levels across the four time points under UV-B treatment. (**A**) Cluster 1. (**B**) Cluster 2. (**C**) Cluster 3. (**D**) Cluster 4. (**E**) Cluster 5. (**F**) Cluster 6. *k*-means clustering grouped the expression profiles of the rice metabolite into six clusters. The x-axis shows different UV-B treatment times, and the y-axis depicts the Z-score standardized per metabolite. The membership factor denotes consistency with the trends in metabolite change in each cluster.

We then used the average value of each differentially abundant metabolites at the four time points to analyze their dynamics under UV-B irradiation, forming six dynamic patterns (Table 1 and Supplemental Table S1). Among these, cluster 1 was composed of amino acids and their derivatives, organic acids and their derivatives, and phenylpropanoids, which significantly increased at 6 h of UV-B irradiation and returned to a lower level at 24 h (Figure 2A and Supplemental Table S1). Cluster 2 included amino acids, nucleotides, organic acids and their derivatives, and phenylpropanoids, with higher levels at 12 h followed by a decrease at 24 h (Figure 2B and Supplemental Table S1). The levels of certain amino acids and derivatives, organic acids and their derivatives, phenylpropanoids, and vitamins consistently decreased within 24 h of UV-B irradiation in cluster 3 (Figure 2C and Supplemental Table S1). Cluster 4 consisted of amino acids, organic acids and their derivatives, and phenylpropanoids, which significantly decreased at UV-B 6 h and UV-B 12 h and remained at a low level at UV-B 24 h (Figure 2D and Supplemental Table S1). Interestingly, the metabolite content of cluster 5, including amino acids and phenylpropanoids, had lower levels at UV-B 6 h and UV-B 12 h, but began to increase significantly at UV-B 24 h (Figure 2E and Supplemental Table S1). However, the metabolites in cluster 6 mainly comprised amino acids and phenylpropanoids, with their levels consistently showing an increase within 24 h of UV-B irradiation (Figure 2F and Supplemental Table S1). These results indicate that UV-B treatment has different effects on the accumulation pattern of different metabolites.

### 2.3. Analysis of Different Metabolites in Rice Leaves Treated with Different UV-B Duration

**Figure 3:**
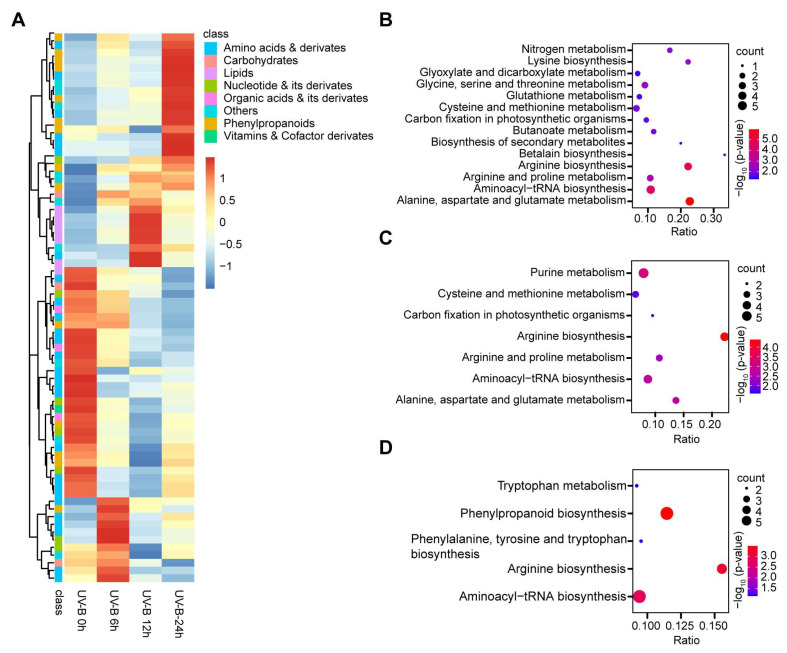
Comparative analysis of metabolome under different UV-B treatment duration in rice. (**A**) Heat map of differential metabolomes under different UV-B treatment durations. Red indicates a high abundance, and blue indicates low relative abundance metabolites. (**B**–**D**) KEGG enrichment of differential metabolites between the comparison groups (UV-B 0 h vs. UV-B 6 h/12 h/24 h). Each bubble in the plot represents a metabolic pathway whose abscissa and bubble size jointly indicate the magnitude of the impact factors of the pathway. The bubble colors represent the *p*-values of the enrichment analysis, with a red color showing a higher degree of enrichment.

To further investigate the effects of UV-B irradiation on metabolites in rice leaves, we analyzed the metabolic pathways of screened different metabolites, using the KEGG to analyze genes expression information and metabolite accumulation. In this study, different metabolites were identified based on the duration of UV-B treatment, and these metabolites were classified according to their pathways. With the different time of UV-B treatment, the content changes of these metabolites were visualized in a heat map (Figure 3A). The enrichment of metabolic pathways was mainly related to amino acids’ metabolism under UV-B 6 h (Figure 3B, Supplemental Table S2) and UV-B 12 h (Figure 3C, Supplemental Table S2) treatments, while phenylpropanoid biosynthesis was enriched in the UV-B 24 h (Figure 3D, Supplemental Table S2) treatments. These results suggest that UV-B mainly affects amino acids metabolism and phenylpropanoid biosynthesis.

### 2.4. UV-B Treatment Alters the Expression Levels of OsbZIP18 in Rice

**Figure 4:**
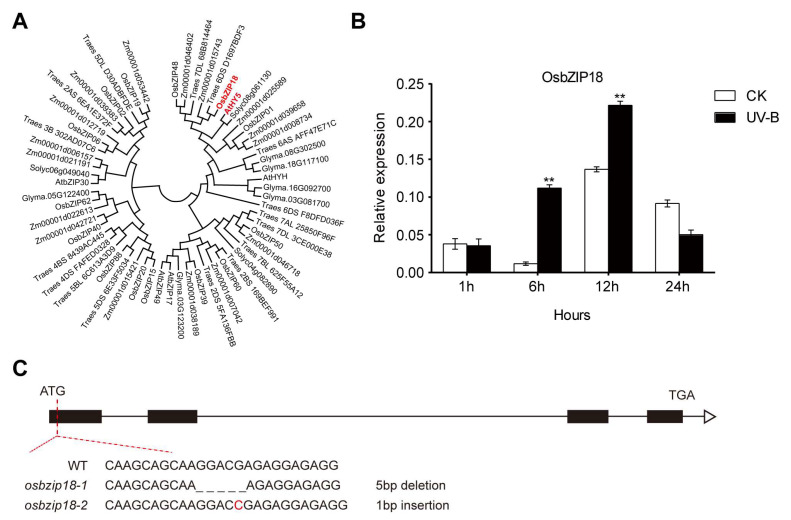
Effects of UV-B treatment on transcription levels of *OsbZIP18* in rice. (**A**) Phylogenetic tree of HY5 protein in *Arabidopsis*, rice, maize, wheat, soybean, and tomato, with the bootstraps values from 1000 replicates indicated. (**B**) Expression analysis of *OsbZIP18* under control and UV-B treatments. The relative expression levels were normalized to the ubiquitin gene and were quantified by RT-qPCR. Asterisks indicate a significant difference between the control group and UV-B treatment at individual time points (n = 3, ** *p* < 0.01, Student’s *t*-test). (**C**) Identification of *osbzip18* mutants. The *osbzip18-1* is 5 bp GGACG deletion and the *osbzip18-2* is 1 bp C insertion in the first exon of *OsbZIP18* genome.

To understand the impact of core transcription factors in the UV-B signaling pathway on metabolites, we decided to analyze the homologues of *AtHY5*. The phylogenetic tree of the HY5 protein in *Arabidopsis*, rice, maize, wheat, soybean, and tomato indicates that OsbZIP18 shares the highest sequence similarity with AtHY5 compared to the others (Figure 4A). Subsequently, we detected the expression of *OsbZIP18* under UV-B stress. The transcript levels of *OsbZIP18* showed a significant increase at 6 h and 12 h after UV-B treatment (Figure 4B). These results suggest that *OsbZIP18* may play a crucial role in the UV-B response in rice.

### 2.5. Analysis of Differential Metabolites of Osbzip18 Mutants Treated with Different UV-B Duration

To determine the function of OsbZIP18 in rice, we generated *osbzip18* CRISPR (*osbzip18*-1 and *osbzip18*-2) lines in the *japonica* cultivar Zhonghua11 (ZH11) background. *Osbzip18-1* carried a deletion of five bases and *osbzip18-2* had an insertion of one base at the target site, which truncated the open reading frame of *OsbZIP18*.

**Figure 5:**
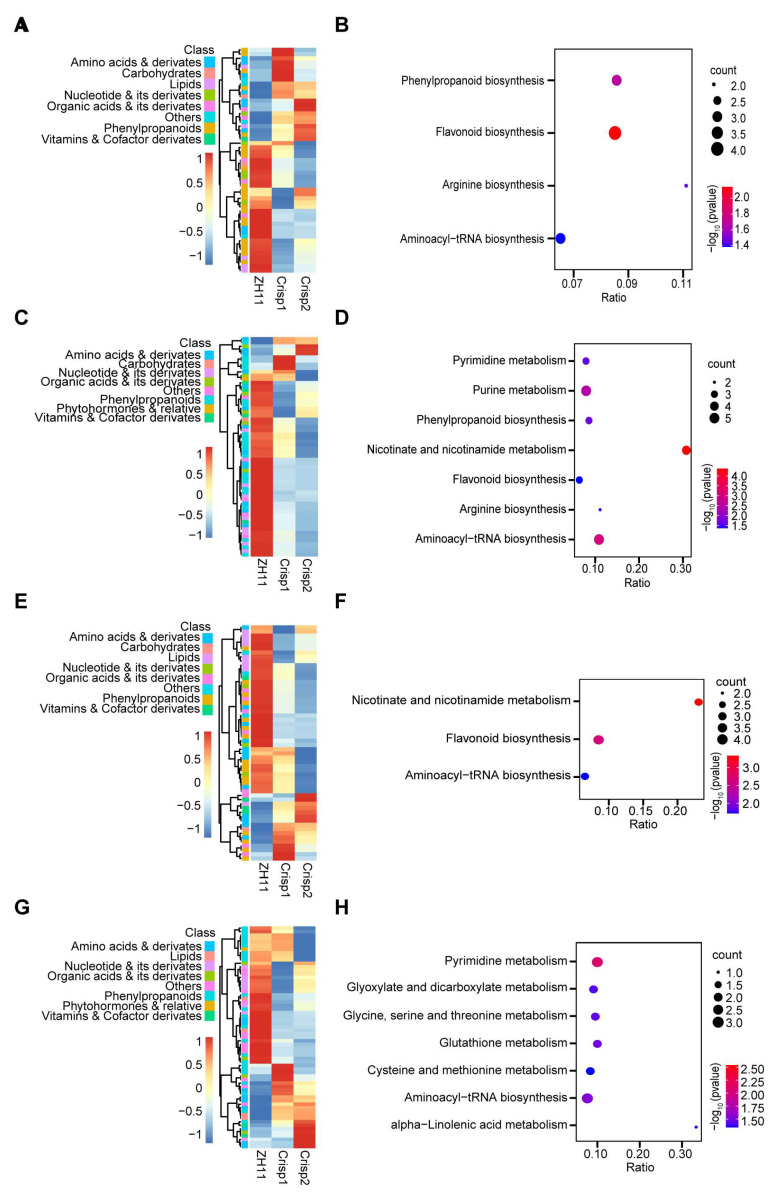
Comparative analysis of differential metabolites of *osbzip18* mutants under different UV-B durations. Heat map of differential metabolites of *osbzip18* mutants under different treatment duration, UV-B 0 h (**A**), UV-B 6 h (**C**), UV-B 12 h (**E**), and UV-B 24 h (**G**). Red indicates a high abundance, and blue indicates low relative abundance metabolites. KEGG pathways enriched significantly in *osbzip18* mutant vs. ZH11 comparison under different treatment duration, UV-B 0 h (**B**), UV-B 6 h (**D**), UV-B 12 h (**F**), and UV-B 24 h (**H**). The bubble colors represent the *p*-values of the enrichment analysis, with red color showing a higher degree of enrichment.

To understand the changes in metabolism in UV-B signaling caused by OsbZIP18, a comparison was carried out between the *osbzip18* mutant and the wide type under different durations of UV-B treatments (Figure 5A–H). Under the normal condition (UV-B 0 h treatment), 70 different metabolites were screened and displayed on a heat map (Figure 5A). KEGG enrichment analysis was performed on the differential metabolites. Four pathways related to phenylpropanoid biosynthesis, flavonoid biosynthesis, arginine biosynthesis, and aminoacyl-tRNA biosynthesis were significantly enriched (Figure 5B and Supplemental Table S3). Under the UV-B 6 h treatment, the levels of phenylpropanoid and flavonoid were significantly decreased in *osbzip18* mutant plants (Figure 5C). Seven pathways were significantly enriched, including pyrimidine metabolism, purine metabolism, phenylpropanoid biosynthesis, nicotinate and nicotinamide, flavonoid biosynthesis, arginine biosynthesis, and aminoacyl-tRNA biosynthesis (Figure 5D and Supplemental Table S3). For the UV-B 12 h treatment, there were 56 different metabolites in the comparison of ZH11 vs. *osbzip18* (Figure 5E). These metabolites were enriched in the pathways of nicotinate and nicotinamide, flavonoid biosynthesis, and aminoacyl-tRNA biosynthesis pathway (Figure 5F and Supplemental Table S3). The phenylpropanoid and flavonoid biosynthesis pathway were significantly enriched in the comparison of ZH11 vs. *osbzip18* after UV-B exposure for 6 h and 12 h, suggesting that OsbZIP18 played a key role in regulating phenylpropanoid and flavonoid accumulation in rice.

### 2.6. OsbZIP18 Regulates the Expression of Phenylpropanoid and Flavonoid Biosynthesis Pathway Genes under UV-B Duration

**Figure 6:**
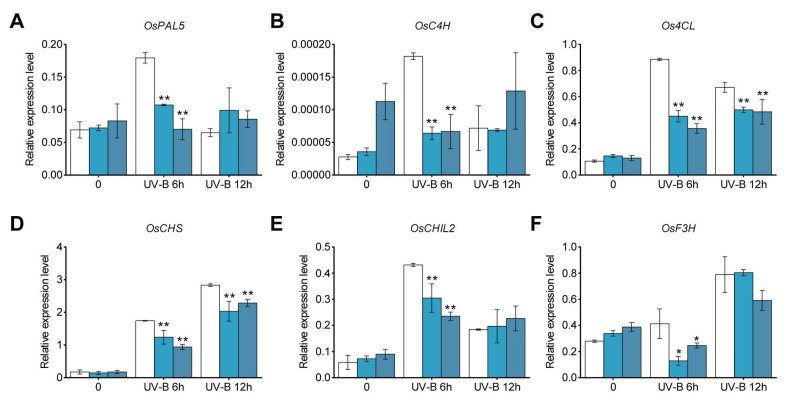
Expression levels of genes involved in phenylpropanoid and flavonoid biosynthesis pathways in ZH11 and *osbzip18* mutants. Abbreviations for enzymes: (**A**) PAL, phenylalanine ammonia lyase; (**B**) C4H, cinnamate 4-hydroxylase; (**C**) 4CL, 4-coumarate CoA ligase; (**D**) CHS, chalcone synthase; (**E**) CHIL2, chalcone isomerase; (**F**) F3H, flavanone 3β-hydroxylase. The relative expression levels were normalized to those of ubiquitin and were quantified by RT-qPCR. Asterisks indicate a significant difference between the control and UV-B treatments at individual time points (n = 3, * *p* < 0.05 and ** *p* < 0.01, Student’s *t*-test).

To investigate whether the expression levels of phenylpropanoid and flavonoid biosynthesis pathway genes were affected in *osbzip18* mutant plants, we performed qRT-PCR analysis. The data showed that the transcription of most genes involved in phenylpropanoid and flavonoid biosynthesis pathways was significantly reduced in *osbzip18* mutants at 6 h, including *OsPAL*, *OsC4H*, *Os4CL*, *OsCHS*, *OsCHI,* and *OsF3H* (*flavanone 3β-Hydroxylase*) (Figure 6). These results indicated that OsbZIP18 positively regulated the expression of phenylpropanoid and flavonoid biosynthesis pathway genes under UV-B duration in rice.

### 2.7. OsbZIP18 Binds to Promoters of OsPAL, OsC4H, Os4CL, OsCHS, OsCHI, and OsF3H In Vitro

**Figure 7:**
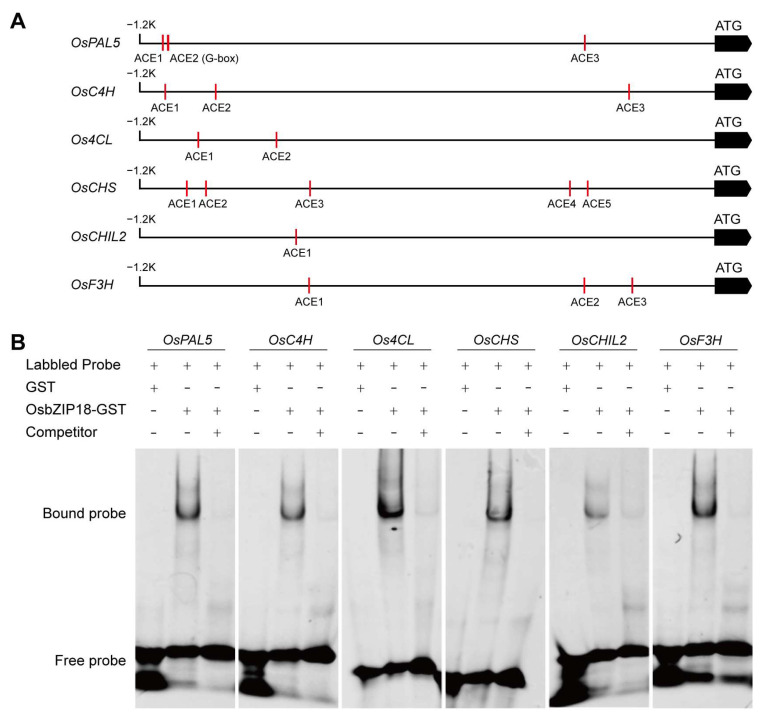
Analysis of cis-binding elements in phenylpropane and flavonoid pathway gene promoter region and electrophoretic mobility shift assays (EMSA). (**A**) Diagram of the *OsPAL5*, *OsC4H*, *Os4CL*, *OsCHS*, *OsCHIL2*, and *OsF3H* promoter regions showing the relative positions of the ACE and G-box cis-elements. The red rectangles represent the ACE elements and the G-box. (**B**) EMSA analysis of OsbZIP18 binding to the ACE and G-box motif in the *OsPAL5*, *OsC4H*, *Os4CL*, *OsCHS*, *OsCHIL2*, and *OsF3H* promoters. Twenty-five-fold molar excesses of unlabeled probes were used in the competition assay.

Recent research showed that OsbZIP18 regulates branched chain amino acid and serotonin synthesis by binding directly to the ACE and C-box cis-elements in the promoters of biosynthetic genes. There was at least one or more ACE-containing or G-box (CACGTG) element within the 1.2 kb region upstream of the transcription start site of the *OsPAL*, *OsC4H*, *Os4CL*, *OsCHS*, *OsCHIL2,* and *OsF3H* genes (Figure 7A). To investigate whether OsbZIP18 could bind to the promoters of these genes in vitro, an EMSA assay was performed. The results showed that OsbZIP18 binds directly to the promoters of *OsPAL5*, *OsC4H*, *Os4CL*, *OsCHS*, *OsCHIL2,* and *OsF3H* (Figure 7B). These results suggested that OsbZIP18 regulated the expression of phenylpropanoid and flavonoid biosynthesis genes by binding to their promoters.

## 3. Discussion

In this study, we identified six patterns of metabolic changes under sustained UV-B stress and revealed that OsbZIP18 is a key regulator of phenylpropanoid and flavonoid biosynthesis by comparing the metabolome at different time points under UV-B stress.

Metabolic adjustments play a significant role in plant adaptation to different abiotic stresses [30]. UV-B is an important environmental signal perceived by plants to regulate plant growth and development. Prolonged exposure to UV-B may increase the production of reactive oxygen species (ROS) [2]. To adapt to UV-B stress condition, plants accumulate many protective metabolites such as flavonoids, hydroxycinnamic acid esters, carotenoids, and vitamin C to minimize the harmful effects [51]. Fox example, *Arabidopsis* mutants that do not accumulate flavonols are highly sensitive to UV-B, whereas the overaccumulation of flavonols in Arabidopsis and rice enhances tolerance to UV-B. Flavonoids, along with other phenylpropanoids, are synthesized from phenylalanine [54]. In this study, we found that the levels of many flavonoids, such as apigenin, luteolin, eriodictyol, kaempferol, and quercetin, with glycosylation increased significantly after UV-B irradiation. Phenylpropanoid biosynthesis and phenylalanine, tyrosine, and tryptophan biosynthesis were significantly enriched under UV-B stress (Figure 2 and Figure 3). These results suggested that flavonoids are essential for resistance to high levels of UV-B in rice.

Many effects of UV-B on plants involve differential gene expression. Transcriptomic analyses with maize [55,56] and *Arabidopsis* [57,58] demonstrate that UV-B regulates numerous genes concerned with various cellular processes. In *Arabidopsis*, HY5 acts as a central regulator of UV-B protection, promoting photomorphogenesis downstream of multiple photoreceptors and initiating the expression of light-induced genes [59]. HY5 induces the expression of flavonoid biosynthetic genes under both visible and UV-B light, resulting in flavonoid accumulation [44,45,60,61]. HY5 and HYH play a complementary role in regulating flavonoid biosynthesis in the vegetative and reproductive organs of Grapevine (*Vitis vinifera*) after the UV-B stimulus [62]. In rice, three homologs of *AtHY5* (*OsbZIP01*, *OsbZIP18*, and *OsbZIP48*) have been identified, and all were induced by UV-B radiation [52,63,64]. *OsbZIP18* and *OsbZIP48* can respond to UV-B stress, but *OsbZIP18* positively regulates the accumulation of serotonin by regulating the biosynthesis pathway genes *OsTDC* and *OsT5H* and improves the sensitivity to UV-B stress [53]. *OsbZIP48* regulates the accumulation of flavonoids by controlling the gene expression of the flavonoid biosynthesis pathway, thereby improving the tolerance to UV-B stress [50]. In addition, *OsbZIP48* is activated by the phosphorylation of protein kinase RLCK160 to regulate the accumulation of flavonoids, but *OsbZIP18* has not been found to be regulated by other kinases. In our study, our results revealed that the loss of OsbZIP18 impairs phenylpropanoid and flavonoid biosynthesis under UV-B treatment. 

Flavonoids constitute one of the most abundant groups of secondary metabolites in plants and are synthesized via the phenylpropanoid pathway. General phenylpropanoid metabolism starts with phenylalanine and involves the activity of three enzymes: phenylalanine ammonia lyase (PAL), cinnamate 4-hydroxylase (C4H), and 4-coumaroyl CoA ligase (4CL) [65]. Together, these enzymes produce p-coumaroyl CoA, which acts as the activated intermediate for various branches of phenylpropanoid metabolism [66,67]. P-Coumaroyl-CoA is condensed with three molecules of malonyl-CoA to naringenin chalcone by chalcone synthase (CHS), which is then converted to the flavanone naringenin by chalcone isomerase (CHI) [68,69,70]. The transcription levels of genes involved in the phenylpropanoid and flavonoid biosynthesis pathway, such as *OsPAL*, *OsC4H*, *Os4CL*, *OsCHS, OsCHIL2,* and *OsF3H*, were decreased in *osbzip18* mutants (Figure 6). Additionally, the levels of flavonoids, including kaempferol, eriodictyol, quercetin, apigenin, luteolin, and their C-glycosylated or O-glycosylated derivatives, showed a decreasing trend in *osbzip18* mutants under UV-B irradiation. Therefore, our results indicated that OsbZIP18 functions as a positive regulator of phenylpropanoid and flavonoid biosynthesis under UV-B duration, which mediated phenylpropanoid and flavonoid accumulation by modulating the key biosynthetic genes, suggesting an instance of divergent evolution of gene functions. In summary, we demonstrated that *OsbZIP18* plays a crucial role in regulating phenylpropanoid and flavonoid biosynthesis under the UV-B stress in rice.

## 4. Materials and Methods

### 4.1. Plant Materials and Growth Conditions

The japonica rice variety Zhonghua 11 (ZH11) was used as the wild type. The *osbzip18* mutants were obtained using the CRISPR-Cas9 method as previously described [53]. *Crispr-1* is 5 bp GGACG deletion and *crispr-2* is 1 bp C insertion in the first exon of *OsbZIP18* genome. Seeds of the *osbzip18* mutants and ZH11 were germinated for 3 d at 37 °C on filter paper soaked with distilled water. After germination, the seeds were transferred to a net floating on distilled water in a greenhouse for 7 d. The seedlings used in the following experiments were transferred to a plastic container for hydroponic culturing in a greenhouse. The nutrient solution contained 1.43 mM NH_4_NO_3_, 0.32 mM NaH_2_PO_4_, 0.51 mM K_2_SO_4_, 1.00 mM CaCl_2_, 1.64 mM MgSO_4_, 0.17 mM Na_2_SiO_3_, 16 µM EDTA-Fe (II), 0.075 µM (NH_4_)_6_Mo_7_O_24_, 19 µM H_3_BO_3_, 9.47 µM MnCl_2_, 0.16 µM CuSO_4_, 0.15 µM ZnSO_4_, 35.61 µM FeCl_3_, and 70.78 µM citric acid (pH 5.0), which was renewed every 3 d. 

For UV-B experiments, rice seedlings were cultivated in a plant growth chamber with supplemental narrowband UV-B. The intensity of UV-B was measured at 3.2 mW/cm^2^, using a UV radiometer equipped with a UV-295 detector from the photoelectric instrument factory of Beijing Normal University, China. Philips, Netherlands, TL8W/302 nm narrowband UV-B tubes were used, as mentioned in a previous study [53]. The control group did not have UV-B lamps installed. Then, the leaves of two-week-old seedlings were collected separately with three biological replications to determine the metabolite content as previously described [53]. Each biological replication was mixed with eight different plants.

### 4.2. Metabolites Extraction, Detection and Analysis

The freeze-dried leaves were crushed using a mixer mill (MM 400; Retsch, Haan, Germany) with a zirconia bead for 45 s at 30 Hz. Then, 100 mg of the dry powder were extracted overnight at 4 °C with 1 mL of 70% aqueous methanol (methanol: H_2_O, 7:3, *v*/*v*). After centrifugation (4 °C, 10,000 rpm, 10 min) and filtration (SCAA-104, 0.22 µm pore size; Angel, Shanghai, China), the metabolites in the mixture were analyzed using an LC-electro spray ionization (ESI)-MS/MS system as previously described [71,72].

The sample extracts were broadly targeted for detection by the QTrap system (QTRAP 6500+; AB Sciex). The detection results were then compared with the standard and the database, and a total of 308 metabolites were identified. For the QTrap system, the ESI source parameters were set as follows: the temperature at 500 °C, GSI at 50 psi, GSII at 60 psi, CUR at 35 psi, and IS at 5500 V in positive mode or −4500 V in negative mode. The collision gas used was high in the schedule multiple reaction monitoring (sMRM) scan mode. The total cycle time for sMRM was set to 0.8 s, and the dwell time for each MRM transition was automatically adjusted in accordance with the total cycle time. This ensured that each peak had a minimum of 10 points. The mass raw data obtained were processed using MultiQuant 3.0.3 software, and the peak areas were integrated using the MQ4 integration algorithm.

The quantification of metabolites was carried out using a scheduled multiple reaction monitoring (MRM) method, with an MRM detection window of 80 s and a target scan time of 1.5 s. The differential metabolites were screened out by combining VIP (Variable Importance in the Projection) of OPLS-DA model ≥ 1, log_2_|fold change| ≥ 1, and *p* < 0.05 (Student’s *t*-test).

### 4.3. Principal Component Analysis and KEGG Enrichment

The Factoextra R package was used for principal component analysis of metabolites. KEGG pathway enrichment analysis of the differentially abundant metabolites was performed using MetaboAnalyst 4.0 (http://www.metaboanalyst.ca/ accessed on 20 June 2022). The model organism selected was *Oryza sativa* L. ssp. *Japonica*. The metabolome view was visualized by ggplot2 in R [73], with the adjusted *p* values and pathway impact values arranged on the Y and X axes, respectively.

### 4.4. Phylogenetic Analysis

The amino acid sequences in this study were extracted from the NCBI (https://www.ncbi.nlm.nih.gov/ accessed on 26 June 2022). Multiple-sequence alignments were performed with ClustalW (v.1.83) program, and phylogenetic analysis was conducted by MEGA7 (http://megasoftware.net/ accessed on 25 June 2022) using the neighbor-joining method with 1000 bootstrap replications. 

### 4.5. RNA Extraction and Expression Analyses

The samples of wild type ZH11 and *osbzip18* mutants were collected separately with three biological replications to determine the expression level. RT-qPCR was performed using total RNA extracted with the TransZol RNA Extraction Kit (TransGen Beijing, China). Three micrograms of RNA were used to synthesize the first-strand cDNAs in 20 μL of reaction mixture using EasyScript One-Step gDNA Removal and cDNA Synthesis SuperMix (TransGen) according to the manufacturer’s instructions. The quantitation of transcript abundance was performed using the SYBR Premix Ex Taq kit (TaKaRa, Tokyo, Japan) on the ABI 7500 Real-Time PCR system (Applied Biosystems, Foster City, CA, USA). The rice UBIQUITIN5 gene was used as the internal reference. The primer sequences are listed in Supplemental Table S4.

### 4.6. Statistical Analysis

The data were analyzed using Microsoft Office Excel 2013 and SPSS 23.0 (SPSS, IBM, Chicago, IL, USA). The results are expressed as means ± SD of at least three independent experiments. The differences among groups were determined using a Student’s *t*-test or a one-way ANOVA.

### 4.7. Accession Numbers

The accession numbers of genes in this article are: *OsbZIP18* (LOC_Os02g10860), *OsPAL* (LOC_Os04g43760), *OsC4H* (LOC_Os05g25640), *Os4CL* (LOC_Os03g05780), *OsCHS* (LOC_Os11g32650), *OsCHIL2* (LOC_Os12g02370), *OsF3H* (LOC_Os03g03034). Sequence data from this article can be found in the Rice Genome Annotation Project website (http://rice.plantbiology.msu.edu/ accessed on 5 January 2023 ) and NCBI (https://www.ncbi.nlm.nih.gov/ accessed on 5 January 2023).

## Figures and Tables

**Table 1:** Distribution of the compounds identified in this study among different clusters.

Compounds	Cluster 1	Cluster 2	Cluster 3	Cluster 4	Cluster 5	Cluster 6
Amino acids and theis derivatives	9	8	12	15	5	14
Carbohydrates	1	2	4	0	1	1
Lipids	11	2	3	2	8	1
Nucleotides and their derivatives	3	5	4	7	3	4
Organic acid	5	9	8	4	6	1
Phenylpropanoids	10	8	20	8	26	12
Vitamins and their derivatives	1	6	4	1	3	2
Others	6	10	13	5	6	9

## Data Availability

All data are available upon reasonable request.

## Supplementary Materials

The following supporting information can be downloaded at: https://www.mdpi.com/article/10.3390/plants13040498/s1. Table S1: The clustering of metabolites in ZH11 leaves under different duration UV-B irradiation. Table S2: KEGG enrichment of different metabolites in ZH11 leaves under UV-B irradiation for different durations. Table S3: KEGG enrichment of different metabolites in *osbzip18* mutant and ZH11 leaves under different durations of UV-B irradiation. Table S4: Primers used in this study.
